# T_H_ Cells and Cytokines in Encephalitogenic Disorders

**DOI:** 10.3389/fimmu.2022.822919

**Published:** 2022-03-07

**Authors:** Sinduya Krishnarajah, Burkhard Becher

**Affiliations:** Institute of Experimental Immunology, University of Zurich, Zurich, Switzerland

**Keywords:** helper T (TH) cells, neuroinflammation, cytokines, multiple sclerosis, EAE (experimental autoimmune encephalitis), GMCSF, granulocyte macrophage colony-stimulating factor

## Abstract

The invasion of immune cells into the central nervous system (CNS) is a hallmark of the process we call neuroinflammation. Diseases such as encephalitides or multiple sclerosis (MS) are characterised by the dramatic influx of T lymphocytes and monocytes. The communication between inflammatory infiltrates and CNS resident cells is primarily mediated through cytokines. Over the years, numerous cytokine networks have been assessed to better understand the development of immunopathology in neuroinflammation. In MS for instance, many studies have shown that CD4^+^ T cells infiltrate the CNS and subsequently lead to immunopathology. Inflammatory CD4^+^ T cells, such as T_H_1, T_H_17, GM-CSF-producing helper T cells are big players in chronic neuroinflammation. Conversely, encephalitogenic or meningeal regulatory T cells (T_REGs_) and T_H_2 cells have been shown to drive a decrease in inflammatory functions in microglial cells and thus promote a neuroprotective microenvironment. Recent studies report overlapping as well as differential roles of these cells in tissue inflammation. Taken together, this suggests a more complex relationship between effector T cell subsets in neuroinflammation than has hitherto been established. In this overview, we review the interplay between helper T cell subsets infiltrating the CNS and how they actively contribute to neuroinflammation and degeneration. Importantly, in this context, we will especially focus on the current knowledge regarding the contribution of various helper cell subsets to neuroinflammation by referring to their helper T cell profile in the context of their target cell.

## T Cell Polarisation: An Overview

T cell mediated immunity is reliant on the differentiation of naïve T cells into their effector T cell counterparts. Upon activation, these cells bifurcate into their two major lineages – CD8-expressing cytotoxic T lymphocytes (CTL), and CD4-expressing helper T cells (T_H_) (1). CD4^+^ cells are important in the regulation of the adaptive immune response against a plethora of pathogens. Through differentiation and the secretion of cytokines, these cells help activate antigen-specific B cells to produce antibodies, and hence drive humoral immunity.

About 4 decades ago, it was postulated that CD4 T cells can differentiate into subsets with characteristic effector functions (2). Effector T cells are classified and differentiated based on i) the type of pathogen that elicited the activation and ii) the subsequent group of cytokines secreted by these cells. The main effector subsets of CD4 T cells were historically described to only bifurcate into two distinct populations, driven by their inflammatory milieu (3). Briefly, type 1 versus type 2 immunity was grossly classified as immune responses towards intracellular pathogens versus extracellular parasites and helminths. However, this historical classification has now been revised to include many further helper T cell subsets extending beyond the scope of the original T_H_1 and T_H_2 cells.

Further Helper T cell subsets include T follicular helper (T_FH_) and Regulatory T (T_REG_) cells. T_FH_ cells work alongside T_H_1, T_H_2, or T_H_17 cells to help B cells generate class-switched immunoglobulins of different isotypes, which are recognised by different innate immune effector cells through cell characteristic expression of cell surface Fc receptors. T_REG_ cells, characterised by their expression of the IL-2 receptor alpha chain CD25 (4) alongside with the transcription factor (TF) FoxP3 (5), have immunoregulatory functions and promote tolerance towards the antigens they recognise, usually self-antigens.

**Figure 1:**
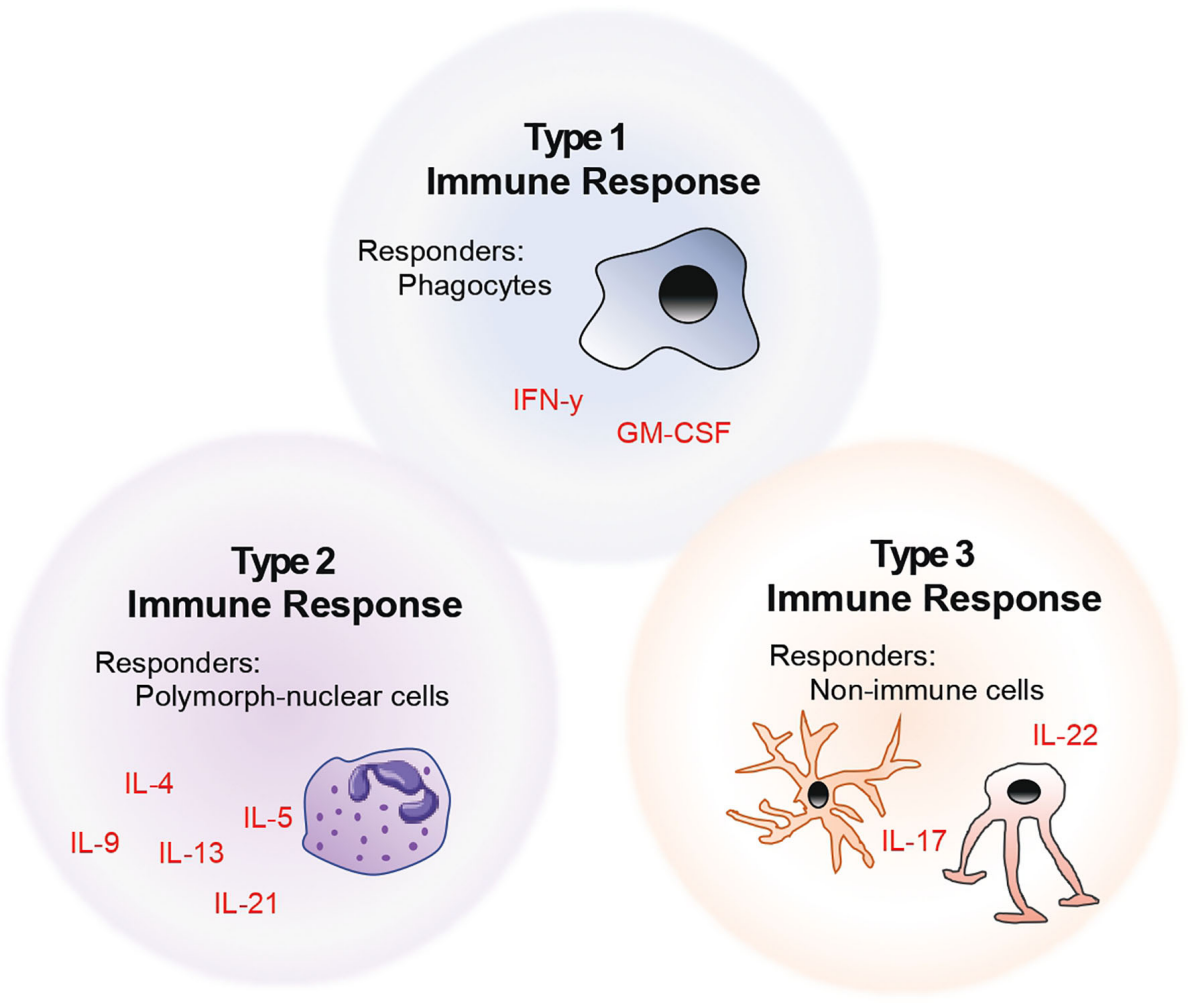
Model of helper T cell classification by considering the role of helper T cells in the context of their target and effector cells.

The above-mentioned descriptions of helper T cell subsets fit the historical classification. However, with increasing advances in the field of immunophenotyping, it has become clear that helper T cell nomenclature in the context of a single lead effector cytokine fails to capture the functional diversity of these cells. Thus, we and others propose that T cells should be rather categorised into the kind of help that these cells provide at a site of injury – based on whether their downstream functions affect i) phagocytes (henceforth referred to as type 1 immunity), ii) polymorph-nucleated cells (type 2), or iii) non-immune cells (type 3) (6). This model of naming and classifying T cells is summarised in the form of a schematic as seen in **Figure 1**. Taking this into account, in this review, we describe the role of helper T cells in the context of their target and effector cells in neuroinflammation.

## Type 1 T_H_ Cells and Neuroinflammation

T_H_1 cells are the most prominent members of the type 1 T_H_ cell family. T_H_1 cells were first characterised by their ability to produce interferon gamma (IFN-γ), a potent cytokine with important immunomodulatory functions. T_H_1 cells help orchestrate the adaptive immune response against intracellular pathogens (e.g. viruses) through direct activation of phagocytic cells or CTLs. These cells in turn directly kill the pathogen or virus infected or transformed host cell in question and can further promote antibody-dependent cellular cytotoxicity (ADCC) and opsonisation.

In addition to IFN-γ, T_H_1 cells can also be recognised by their cell surface expression of the IL-12 receptor (R) β chains (1 and 2) and the chemokine receptor type 3 (CXCR3). Further work from the late 20^th^ century revealed that there are also key TFs which play important roles in T_H_1/T_H_2 polarisation – and thus T-bet was associated with T_H_1, and GATA-3 with T_H_2 cells (7–9). The T_H_1 signal is self-regulating through a positive feedback loop, as IL-12 and IFN-γ both induce T-bet, which in turn induces IFN-γ and T-bet, too (10).

Early studies in an animal model of multiple sclerosis (MS), termed experimental autoimmune encephalomyelitis (EAE), showed that IFN-γ positive cells were the biggest immune cell population in the diseased brain (11, 12), suggesting that T_H_1 cells were potentially very important in the neuro-pathogenesis of the disease. Furthermore, the adoptive transfer of T_H_1 cells into naïve animals was shown to drive neuroinflammation, further supporting this notion (13).

The exact role of these brain-infiltrating CD4^+^ T cells in the context of neuroinflammatory disease is still under investigation. However, a potential downstream target of T_H_1 mediated effector functions in the central nervous system (CNS) are the resident macrophages of the brain called microglia. Like most other resident macrophages of the body, several studies have suggested that T_H_1 cells secreting their signature cytokine cocktail leads to the activation of microglia into an inflammatory phenotype (14). In the parenchyma of the brain, microglia are the only resident leukocytes, which makes them a solid contender to interact with T cells invading the CNS in neuroinflammatory conditions (15).

The capacity of these cells to present antigens has been shown in several *in vitro* studies (16–19). Subsequently, several follow-up studies suggested that microglial activation is directly linked to immune infiltration of the CNS and the maintenance of encephalitogenicity during the effector phase of EAE (20–22). However, in the non-inflamed brain, most cell types including microglia do not express MHC class II or costimulatory molecules. This makes them unlikely to be responsible for the initial reactivation of encephalitogenic T cells.

Key studies were carried out to investigate the *bona-fide* antigen presentation capabilities of CNS-resident cells, using mouse models where MHC class II expression could be restricted to certain antigen presenting cell (APC) subsets. These experiments revealed that *in vivo*, neither microglia, nor any other parenchymal elements are required to mediate interactions between APCs and helpers T cells (23). Building on these findings, systematic interrogation of each potential APC within the brain revealed that among the conventional dendritic cell (cDC) subsets, cDC2s in particular are powerful APCs in bridging CNS-T cell interactions (24). Whilst microglia may not be the main players in initiating neuroinflammatory pathology, it is however feasible that during the chronic phases of the disease, microglia play a role in chronification and disease perpetuation.

The most likely immune cell target for type 1 cytokines such as IFN-γ is in fact not resident to the CNS, but instead may invade the CNS from the circulation, namely monocytes. In mice and humans, monocytes come in two flavours. One that is patrolling in the blood (in mice, Ly6C^low^) and another capable of reacting to inflammatory stimuli and invading tissues (Ly6C^high^). IFN-γ has been shown to be important for the monocyte to macrophage transition in inflamed sites (25). Nevertheless, the functional consequences of this IFN-γ induced maturation of monocytes remain unclear.

Further studies in animals revealed the extent of the role of T_H_1 cells in neuroinflammation. IFN-γ is heavily present in the brain lesions present in EAE mice, and the same holds true for MS patients. Clinical trial data revealed the IFN-γ administration to patients suffering with MS made their symptoms worse, and led to increased relapses (26). In contrast though, mice lacking the IL12R β2 chain (27, 28), or the p35 subunit (29), are susceptible to EAE. The same holds true for animals deficient in IFN-γ (30). Moreover, IL-12 administration to mice suffering from early stages of EAE suppressed the disease – the authors of this study also showed that this was an IFN-γ dependent phenomenon (28). Whilst the majority of historical evidence points towards an overall pathogenic role for IFN-γ producing T_H_1 cells (31), many contradictory studies reveal a potential protective role of these same cells in neuroinflammation (32, 33). To date, the mechanisms by which IL-12 and IFN-γ regulate or suppress neuroinflammation remain completely unknown.

## Type 3 T_H_ Cells and Neuroinflammation

In the context of autoimmunity, studies revealed that IL-23, a cytokine with a shared p40 subunit with IL-12 (34), is important in driving inflammation in models of multiple sclerosis and psoriasiform inflammation. Additionally, the IL-23R comprises the IL12R β1 chain (35) – and these observations helped to clarify the contradictory data described in the previous section. It was then established that IL-23 is a driver of neuroinflammation by the induction of a subset of helper T cells which secrete IL-17 and therefore also activate a type 1 response (36, 37).

Hence, the way was paved for the coining of T_H_17 cells (36). T_H_17 cells produce the cytokines IL-17A, IL-17F, IL-21 and IL-22 as lead cytokines (38). The cells are further characterised by the expression of CCR4, CCR6, CD161 as well as IL23R and IL-1R. In addition, these cells express retinoic acid receptor-elated orphan nuclear receptor γτ (RORγτ) intracellularly.

The main reason we call these cells type 3 immune cells is because their primary targets are non-immune cells. Receptors for IL-17 and IL-22 are expressed in various densities throughout the immune as well as stromal compartments. Dysregulation of IL-17 for instance, leads to inflammation of tissues of the body lining, rich in epithelial cells (39). While these mice developed severe skin inflammation, most solid tissues including the CNS were unaffected. In line with this, dysregulation of any members from this group of cytokines, such as IL-17A/F, IL-21 or IL-22, generally leads to pathologies restricted to barrier tissues, like the skin, lung or gut (40–42).

IL-21 was initially described to play an important role in encephalitogenicity (43) – however, this claim was rebuked by many follow-up studies (44, 45).

Whilst these responses are important to curb off an imminent infection, the flipside of a sustained T_H_17 response is tissue inflammation and damage. In neuroinflammation specifically, these cells have been described to be involved in the pathogenesis of EAE and MS. There have been claims that helper T cells which secrete IL-17 are abundant in both the peripheral blood as well as the cerebrospinal fluid (CSF) of MS patients (46). However, overall, there is no evidence of overt dysregulation of IL-17 signalling itself in MS. Even though a clinical trial neutralising IL-17 in MS has shown some early signs of efficacy, it has not been pursued further and approval was never sought for (47).

Even though disease progression and active disease have also been linked with the increased presence of T_H_17 cells in patients, the most likely contribution from IL-17 in neuroinflammation may be its effects on the blood brain barrier (BBB). Evidence links IL-17 with barrier function in other organs such as the lung and gut (48, 49), with further experimental data pointing towards IL-17 playing a role in altering of the neurovascular junction being convincing (50, 51). In addition, T_H_17 cells from patients in relapsing MS are associated with inflammatory lesions and have increased migratory capacities (52).

Astrocytes are a potential neurological cell type which has been investigated in recent years as an effector cell of T_H_17 responses. They are a subtype of glial cells which reside between the BBB and resident brain cells, are characteristically histologically star-shaped (53), and perform a vast range of functions including tissue maintenance, repair, and regulating cerebral flow. Their main function is directly linked to their location within the brain, where they can monitor and regulate the exchange between the CNS and the systemic circulation (54). Increased expression of a functional IL-17 receptor was demonstrated *in vitro* (55), as well as under EAE conditions (56, 57). Disruption of IL-17 signalling in these cells was shown to improve EAE in mice (58). However, the signalling pathway targeted in these studies is by no means IL-17 specific, and thus the contribution of IL-17 *via* astrocytes towards neuroinflammation remains a subject of debate.

Finally, IL-17 also has an effect on a final CNS resident cell type, known as oligodendrocytes. These cells assemble myelin, which is a multi-layered sheath of lipidous membrane around axonal segments. Studies have shown that T_H_17 cells interfere and inhibit the maturation cycle as well as the survival rate of oligodendrocytes (59, 60).

## Helper T Cell Subsets – Highly Plastic?

As discussed previously, recent mounting evidence has led to the belief that helper T cell subsets may not be rigid and cemented in their functional and expression profiles, but that they may adapt according to environmental cues. This is at least true for T_H_17 cells. There is a strong propensity for these to differentiate into cells that secrete IFN-γ or play the opposing role by producing non-inflammatory IL-10 (61).

A study by Capone and colleagues demonstrated this principle. In relapsing MS patients, T_H_17 cells upregulate the expression of IL-1R and produce higher levels of IL-21,IL-2, and TNF-β (62). Similarly, within the T_H_17 compartment of MS patients with active symptoms, another study found elevated expression of IFN-γ and CXCR3 together with reduced expression of IL-10 (63). Conversely, T_REGs_ have been shown to be highly stable (64).

Recent studies have gone a step further and suggested the notion that these subsets may be overlapping in such a manner that their current naming is largely redundant. Cells that secrete both IFN-γ as well as IL-17, hence sitting on the fence between a T_H_1 and T_H_17 phenotype (65, 66), have been reported on several occasions. These cells express the receptor for IL-23R. In addition, they co-express CXCR3 and T-bet together with CCR6 and RORγτ. Interestingly, they have been described to produce lower amounts of IL-17A compared to classical T_H_17 cells but high levels of IFN-γ [reviewed in (67)]. Specifically, in the context of neuroinflammation, cells characterised by the expression of TNF, IFN-γ, IL-2, the CXC chemokine receptor type 4 (CXCR4) and very late antigen 4 (VLA4) were convergent in the blood of patients with MS. These cells were also enriched within the CNS, and were drastically reduced upon therapeutic intervention (68). During acute EAE, cells with a similar mixed helper T cell phenotype can cross the BBB and accumulate in the CNS. Finally, cells with a similar phenotypic profile were also found in brain tissues from MS patients and upregulated in patients during relapse (69, 70).

The observed plasticity across TH cells is clearly beneficial to immunity in the fight against infections. An overly rigid, hard-wired program makes little sense given that the primary role of T_H_ cells is providing ‘help’. This is why we believe that, in the future, a categorisation based on single cytokines or even multiple cytokines will fade in favour of a more nimble and logical description across their specific helper function (6).

## GM-CSF: Licensing of Phagocytes for Immunopathology

In line with a categorisation of T_H_ cells towards their helper function, another prominent cytokine produced by type 1 T_H_ cells is the granulocyte macrophage colony-stimulating factor (GM-CSF). GM-CSF was originally classified as a growth factor contributing to haematopoiesis upon its discovery, as it was shown to lead to the differentiation of bone marrow progenitors into granulocytes and macrophages *in vitro* (71–73). What makes GM-CSF unique among other CSFs is that lack of either the cytokine, or its receptor, does not lead to any disturbance to myeloid cell development or maintenance in mice (74–76), despite its receptor being almost exclusively expressed within the myeloid compartment.

*In vitro*, there are compelling data to suggest that GM-CSF promotes DC differentiation from both human and mouse progenitor cells (73, 77). However, the same could not be readily replicated *in vivo* (78). What was clear is the role of GM-CSF in tissue inflammation, due to evidence pointing to its role in activation and survival of many myeloid cell subtypes such as neutrophils, monocytes and macrophages (79, 80).

GM-CSF expression originates from a plethora of cell types, including haematopoietic cells as well as epithelial or endothelial cells, fibroblasts and stromal cells. Under steady state, healthy physiological conditions, GM-CSF is rarely detected in physiological conditions *in vivo* – rather, its secretion has also been associated with sites of inflammatory injury (81–83). T_H_ cells secreting GM-CSF were shown to be induced by IL-23 (84), and El-Behi et al. showed that GM-CSF producing cells promote a positive-feedback loop to keep stimulating IL-23 secretion (85). The evidence that GM-CSF is a mandatory cytokine produced by encephalitogenic T cells is overwhelming. IL-1β can further elicit GM-CSF secretion in T_H_17 cells *in vitro*, while IL-27, IFN-γ and IL-12 counteracts GM-CSF production (21, 84).

Using a fate-mapping and reporter system for GM-CSF expressing cells, it was shown that secretion of GM-CSF was both IL-23 and IL-1β dependent (86). The specific role of each of these individual cytokines on the expression of GM-CSF is yet to be elucidated. In the same study, cells that formerly secreted GM-CSF were shown to be more likely to express GM-CSF once again in a recall setting as opposed to their GM-CSF naïve counterparts (86). Another study revealed that antigen-independent GM-CSF release by T_H_ cells, and this cytokine alone, was enough to induce neuroinflammation. Interestingly, whilst GM-CSF lead to severe neurological symptoms, other organs were not affected (87). In this study, the authors showed that GM-CSF-induced infiltration of inflammatory phagocytes was confined to the CNS, liver, and lung. Conversely, the skin, colon, and pancreas were spared. This suggests that the specific tissue microenvironments harbour different cues for the invasion of myeloid cells. In addition, it seems that the microenvironment of the target tissue itself influences the effector function of these cells, since the inflammatory phagocytes found in the CNS had a unique genetic signature when compared to the phagocytes within the other tissues. Microarray analysis of *in vitro*-differentiated cytokine-secreting T_H_ cells identified a large portfolio of genes that were exclusively expressed in GM-CSF-secreting T_H_ cells (88). Altogether, these findings support the notion of a distinct T_H_ subset related to GM-CSF driving neuroinflammation.

## Conclusions

There is no doubt that encephalitogenic T_H_ cells play an important role of in propagating neuroinflammation. Even though there is a heavy debate as to whether MS is primarily driven by type 1 or type 3 cytokines, if one considers the cellular composition within neuroinflammatory lesions, it should be termed a type 1-driven immune response. However, the ability of type 3 cytokines (e.g. IL-17) to interact with epithelial and endothelial cells, suggests a role of type 3 immunity in BBB dysfunction. The interplay of other factors and the rest of the cytokine network in neuroinflammation remains to be established. Currently ongoing research is targeted towards elucidating these unanswered questions. Among the most pressing questions is the relative role of CNS resident versus invading cells in immunopathology, and how this intertwines with the instruction delivered by CNS invading T_H_ cells. Equally, among the biggest challenges will be to identify unique molecular patterns of encephalitogenic T_H_ cells which allows for their targeting and neutralisation without collateral broad immunosuppression.

## Author Contributions

SK conceptualised the manuscript and wrote the first draft. Both SK and BB critically reviewed and revised the manuscript.

## Funding

Funding from the European Research Council (ERC) under the European Union’s Horizon 2020 research and innovation programme grant agreement No 882424, the Swiss National Science Foundation (733 310030_170320, 310030_188450, and CRSII5_183478 to BB), the CRPP Immunocure and the LOOP Zurich.

## Conflict of Interest

The authors declare that the research was conducted in the absence of any commercial or financial relationships that could be construed as a potential conflict of interest.

## Publisher’s Note

All claims expressed in this article are solely those of the authors and do not necessarily represent those of their affiliated organizations, or those of the publisher, the editors and the reviewers. Any product that may be evaluated in this article, or claim that may be made by its manufacturer, is not guaranteed or endorsed by the publisher.
